# Economic claims following chronic pain after inguinal hernia repair

**DOI:** 10.1007/s10029-026-03796-3

**Published:** 2026-07-14

**Authors:** Kenney Fehrenkamp Pedersen, Martin Frimand Rønnow, Thue Bisgaard

**Affiliations:** 1 Department of Medical and Clinical Affairs, Hedia Aps, Copenhagen, Denmark; 2 Department of Clinical Sciences, Lund University, Malmö, Sweden; 3 Department of Surgery, Blekinge County Hospital, Karlskrona, Sweden; 4 Department of Surgery, Centre for Surgical Science, Zealand University Hospital, Køge, Denmark

**Keywords:** Inguinal hernia, Chronic pain, Neuralgia, Outcome, Economic claim compensation

## Abstract

**Purpose:**

Economic claims after surgery may be regarded as an alternative surrogate outcome for long-term deprived quality of life. This study reports economic claims of chronic pain following inguinal hernia repair.

**Methods:**

Consecutive data on economic claims following inguinal hernia repair was collected from the nationwide Danish Patient Compensation Association. Patients’ claims were stratified into three groups: 1) isolated chronic pain claims without claims of competing potential reasons for chronic pain (ICP); 2) diverse claims not involving claims of chronic pain (NCP); and 3) claims involving a combination of chronic pain and competing potential claim reasons for chronic pain (CCP).

**Results:**

A total of 507 patients were included and 256 (50.5%) filed a claim involving chronic pain. Follow-up was 100% and median time from hernia repair to patient filing a claim in the ICP group was 1.5 years (IQR 0.6–2.6 years). ICP, NCP and CCP comprised 172 patients (33.9%), 251 patients (49.5%) and 84 patients (16.6%) respectively. Chronic pain was by far the most common claim reason (33.6% of all claim reasons). The median sum of granted compensation per patient in the ICP, NCP and CCP groups was €14,440 (IQR 7,233–100,600), €6,289 (4,024–12,094) and €7,777 (5,639–11,781) respectively.

**Conclusion:**

Long-term chronic pain alone, not involving other complications, was by far the most common reason for seeking economic compensation. Economic compensation of isolated chronic pain (ICP) was rare, but when awarded, was substantially higher than compensation for other claims.

**Supplementary Information:**

The online version contains supplementary material available at 10.1007/s10029-026-03796-3.

## Introduction

During the last the last 20 years chronic pain has been acknowledged as the dominating postoperative complication after inguinal hernia repair. However, chronic pain after inguinal hernia repair is inconsistently defined in the literature – from vague symptoms to debilitating daily complaints disrupting quality of life. In the majority of publications intensity of chronic pain has been registered using different indirect numeric scales such as the Numeric Rating Scale (NRS), Visual Analogue Scale (VAS), etc. without a clear definition of chronic pain; 10 mm on the VAS or for instance > 80 mm etc. [1]. Furthermore, the impact of postoperative chronic pain in daily quality of life may be clinically challenging to measure as a valid clinical parameter [1]. Economic claims after inguinal hernia repair and other surgical procedures including ventral hernia repair have previously been reported, however chronic pain has not been analysed as the primary outcome [2–8]. Economic claim reasons of chronic pain may potentially be an important outcome after inguinal hernia repair. Ultimately, no previous studies on economic claims after inguinal hernia repair or other surgical procedures have analysed claims of chronic pain in the context of concomitant claim reasons (claims of isolated chronic pain vs. claims not including chronic pain/claims of chronic pain in combination with postoperative complications). Thus, the incidence of economic claims due to isolated chronic pain is not known.

Before the present study it was hypothesised that claims of economic compensation due to chronic pain dominates compared to other types of claims. The primary outcome was number of patients filing a claim of isolated chronic pain as well as other claim reasons associated with chronic pain. The secondary outcome was economic compensation. 

### Methods

This was a consecutive retrospective register analysis of claim data, based on a nationwide closed non-public register, the Danish Patient Compensation Association. Economic claims from a 15-year period (2007–2022) were included. Eligibility criteria, data analyses, outcome definitions and claim reasons were defined pre-study. The nature of the registry secured a 100% follow-up of all patients filing claims after inguinal hernia repair during the inclusion period. 

#### The danish patient compensation association

The Danish Patient Compensation Association administers the Danish Patient Compensation Act [9]. The organisation is responsible for managing claims of economic compensation founded in the Danish healthcare system. The registration is mandatory nationwide with 100% coverage of all economic claims. The patients’ claims, medical records and economic compensation information are stored in the Danish Patient Compensation Association database. Financial compensation following a complaint is awarded based on surgical expert panel evaluation. In general, the Danish Patient Compensation Association evaluates claims based on different criteria. The 2%-rule is the by far most used and accounts for all types of surgical procedures. If the risk of a complication is 2% or less, compensation is awarded. 

#### Statement on compliance with ethical standards

This study was performed as a closed non-public register study (the Danish Patient Compensation Association) and, in accordance with Danish regulations, approval from the Ethics Committee and the Danish Patient Safety Authority was not required. Patients seeking compensation from the Danish Patient Compensation Association provide informed consent to be included in the database.

### Eligibility criteria

Data extraction of claims after inguinal hernia repair was performed based on NOMESCO codes (JAB10–97 and JAC10–97) and ICD-10-codes (K40–40.9 and K41–41.9). Patients 18 years old or older who underwent elective unilateral or bilateral primary or recurrent open or laparoscopic inguinal or femoral hernia mesh-repair in Denmark were included for analysis. Exclusion criteria were patients younger than 18 years, patient double registration, emergency operation, insufficient medical records, diagnosis/operation code mismatch and open suture-repair (<1% in Denmark) to reduce the risk of heterogeneity of the data analysis and thereby reduce the risk of a statistical type II error. 

### Definitions and data collection

All included patients filed one claim consisting of one or several claim reasons. Claim reasons and definitions of postoperative complications were defined pre-study (Table 1). One or more claim reasons (chronic pain together with a surgical complication, cosmetic results, etc.) were allowed per patient. Chronic pain was defined as a patient claim of pain from the groin and/or the testis, persisting more than three months after inguinal hernia repair. Thus, chronic pain was defined as the period from operation to filing a claim of economic compensation. Testicular-related injury was an individual claim reason. It was decided pre-study to analyse data into three different study groups: 1) isolated chronic pain claims without claims of competing potential reasons for chronic pain (ICP); 2) diverse claims not involving claims of chronic pain (NCP); and 3) claims involving a combination of chronic pain and competing potential claim reasons for chronic pain (CCP). Claim data were collected from a standardised claim form from the Danish Patient Compensation Association. The form was filled out by the patients themselves or their proxies (e.g. patients, families or lawyers). Data were extracted from the individual patient’s medical file. An operation for recurrence was a proxy for inguinal hernia recurrence [10]. 

**Table 1:** Pre-study defined claim reasons

Claim reason	Definition
Bleeding	Loss of blood requiring blood transfusion
Chronic pain	Post-operative groin pain lasting > 90 days
Chronic sinus	Non-healing wound persisting > 90 days
Cosmetic	Complaints of the cosmetic outcome but not associated with symptoms
Hernia recurrence	Recurrence of hernia at the same location as originally operated.
Medical & anaesthetic	Complication requiring medical treatment or caused by the anaesthesia procedure
Other	Any complication that does not meet the definition of any other claim reason
Sensory defect	Sensory disturbance
Surgical site infection	Infection of the surgical site requiring debridement
Testicular-related injury	Iatrogenic injury to the testicular vessels/orchitis
Visceral injury	Iatrogenic injury
Wound complications	Hematoma, seroma and surgical site infections not requiring debridement

### Statistics

The study was explorative in nature and sample size calculation was not conducted. A sampling size of 15 years was arbitrarily defined pre-study. Frequency distributions, medians (IQR) or number (%) were reported as appropriate. No significance testing were conducted as group distribution were not deemed relevant for the study’s purpose. 

## Results

A total of 507 patient claims were included for analysis during a 15-year period (2007 2022). Overall, 271 (53.5%) of the 507 patients underwent laparoscopic repair. The number of patients undergoing a laparoscopic repair in the ICP, NPC and CCP groups were 81 (46.1%), 143 (57.0%) and 47 (56.0%) respectively. Gender distribution of patients was predominantly male with 445 (87.8%) vs. 62 female (12.2%). The number of males in the ICP, NPC and CCP groups were 149 (86.6%), 219 (87.3%) and 77 (91.7%) respectively. The median age was 52 years (41–64). The age distribution in the ICP, NPC and CCP groups was 47 years (38–55), 60 years (45–69) and 48 years (40–65) respectively. Follow-up was 100% and median time from hernia repair to patient filing a claim in the ICP group was 1.5 years (IQR 0.6–2.6 years). 

**Fig. 1:**
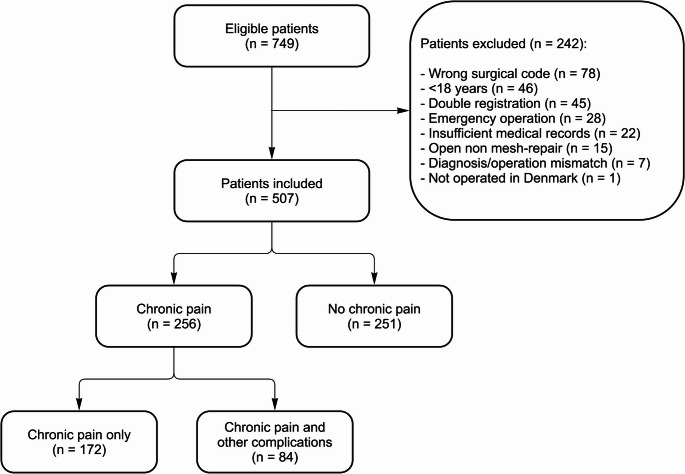
Study profile. A total of 507 patients (n) were analysed after elective inguinal hernia repair. Alt text: “a graphic overview of 749 patients eligible for inclusion, presenting details on 242 excluded patients, and 507 included patients”

**Fig. 2:**
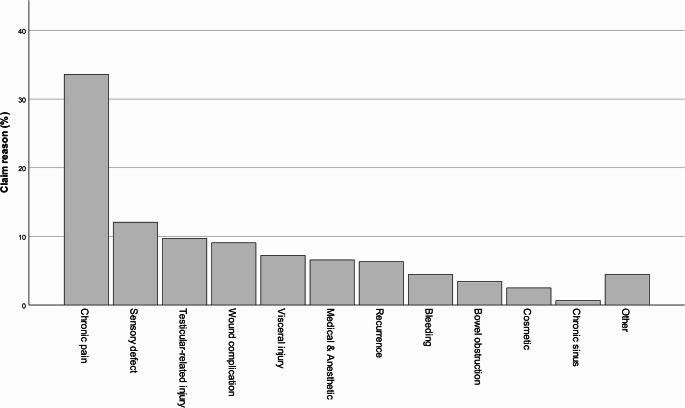
Distribution of total claim reasons (*n* = 762) from 507 patients, as a percentage of all claims. More than one claim reasons per patients was allowed. Alt text: “a bar chart illustrating different claim reasons sorted by percentage of all claims”

Of the 507 included patients 256 (50.5%) filed a claim involving chronic pain. ICP, NCP and CCP comprised 172 patients (33.9%), 251 patients (49.5%) and 84 patients (16.6%) respectively (Fig. 1). The 507 included patients filed a total of 762 claim reasons (Fig. 2). The most common claim reason was chronic pain, with 256 claims (33.6% of all claim reasons), followed by sensory defects, with 93 claims (12.1%), and 74 (9.7%) claims for testicular-related injury. Thus, of the 507 patients, 256 (50.5%) raised an economic claim involving either isolated or chronic pain in combination with other claims (Fig. 1).

A total of 185 patients (36.5%) were granted compensation with a median amount of €7,822 (5,036–15,116). The number of patients granted economic compensation in the ICP, NCP and CCP groups was 36 (20.9%), 110 (43.8%) and 39 (46.4%) respectively. The median sum of granted compensation in the ICP, NCP and CCP groups was €14,440 (IQR 7,233–100,600), €6,289 (4,024–12,094) and €7,777 (5,639–11,781) respectively. The total settlement amount in the ICP, NCP and CCP group was €3,286,616, €1,833,801 and €922,292 respectively. 

### Discussion

In this study isolated long-term chronic pain without competing claim reasons was by far the most common reason for seeking economic compensation. Claims of isolated chronic pain were rarely compensated in comparison with combined claims or claims not involving chronic pain. In the vast majority of chronic pain studies after inguinal hernia repair, patients were followed-up at 3–6 months. However, in the present study a 100% follow-up was achieved with a median of 1.5 years.Groin hernia repair is one of the most common surgical procedures worldwide with approximately 12,000 procedures performed annually in Denmark and 800,000 in the USA [11]. The present study comprised data from 507 patients (one claim per repair) over 15 years. Thus, in Denmark the claim rate was only 0.28% (claims/repair). This suggest that economic claims is a highly selected endpoint. On the other hand, claim data may represent the tip of the iceberg although not supported by the literature. The risk of severe clinically significant chronic pain following groin hernia repair is estimated to be about 3–5% [1]. However, chronic pain is variably defined in the literature, especially concerning the distinction between clinically significant and non-clinically significant chronic pain. Moreover, the vast majority of studies report chronic pain after no more than 3–6 months [1]. As mentioned above, in the present study the follow-up for chronic pain was after median 1.5 years, underlining the chronic status of the pain condition leading to an economic claim. The long follow-up time may stress that chronic pain is a long-term complication beyond one year after inguinal hernia repair. In the present study, chronic pain was the sole patient-reported outcome. Thus, the present study does not provide information on quality of life, numbness in the groin, discomfort etc. Therefore, the present study does not provide the full clinical picture after inguinal hernia repair. 

Economic claim data have previously been analysed after scaphoid fractures [8], primary total hip replacement [5], breast surgery [6] and after umbilical hernia [3], ventral hernia [12, 13] and inguinal hernia repair [2, 4]. In the present study claims of chronic pain, either isolated or in combination with other claim reasons, were found to be three times higher compared to previous reports [2, 4]. The difference may be due to a variety of definitions of chronic pain and follow-up time. Economic compensation in Denmark is partly evaluated based on the risk of the cause of the claim occurring with a <2% probability. The estimated risk of chronic pain is more than 2% (3 – 5%) and the number of compensated claims is probably underestimated in the present study [1]. The present study found that claims of ICP were rarely compensated, but when compensation was awarded patients received a higher compensation compared with NCP or CCP. In a previous retrospective analysis [2] claims of chronic pain following inguinal hernia repair were found to predict an unsuccessful claim outcome in accordance with our findings. Recent analyses of Italian legal malpractice claims following inguinal hernia repair indicate that nerve injury was associated with economic compensation, [14] however comparisons are limited by smaller cohorts and claim evaluation differing significantly from the no-blame system used in Scandinavia (see the Danish Patient Compensation Association above). There are no data comparing the incidence of chronic pain claims compared with claims due to medicolegal malpractice.

In the present study 53.5% of all patients filed a claim following a laparoscopic hernia repair (equally divided between the subgroups). A laparoscopic repair of an inguinal hernia has a lower risk of chronic pain compared with open repair, as supported by our findings [1]. In the present study gender distribution was 445 males and 62 females and claims of chronic pain were comparatively rare in females. Female gender is a known risk factor for chronic pain after inguinal hernia repair, but most inguinal hernia repairs are on male patients, which is in accordance with our findings. Young age is a known risk factor for developing chronic pain [15]. In the present study patients filing a claim of isolated chronic pain had the youngest median age although the subgroup differences were small. 

### Study limitations

This study has several limitations. First, presumably not all patients suffering unsatisfactory outcomes will file a claim for compensation, representing a risk of selection bias. Despite this, claim data are valuable in illustrating patterns in adverse long-term outcomes. Second, preoperative data on chronic inguinal pain were not available in the present study. Thus, claim reasons of chronic pain may be overestimated. Third, compensation after patient claims in Denmark depends on the 2% rule. The number of granted claims would be expected to change given a different percentage rule. Fourth, the present study did not analyse known risk factors for chronic pain (gender, surgical approach, including fixation (glue vs. tacks), age, etc.). However, the present study did not aim to analyse risk factors per se for chronic pain and multivariate analysis was not used. Fifth, the study was based on retrospective patient claims and data was not derived from a prospective clinical trial of chronic pain outcomes after inguinal hernia repair. However, the study was nation-wide and outcomes pre-study defined. Lastly, the analysis was not blinded towards the author performing the data collection, introducing a risk of data registration bias. 

### Future studies

Measuring chronic pain as a claim reason following economic claims represents a clinically significant problem that may only reflect the tip of the iceberg. Thus, an economic complaint of long-term chronic pain after a minor non-acute surgical procedure may potentially be used in future studies as an important surrogate for long-term deprived quality of life. 

## Conclusion

Isolated long-term chronic pain alone after 1.5 years of median follow-up was by far the most common reason for seeking economic compensation. However, economic compensation of isolated chronic pain claims was rare, but substantially higher when awarded in comparison with other types of claims.

## Supplementary Information

Supplementary Material 1.

Supplementary Material 2.

Supplementary Material 3.

## Data Availability

Datasets can be accessed through contact with the corresponding author. The corresponding author had complete access to the study data supporting this paper.
